# Supplementary material for: DeepSLICEM: Clustering CryoEM particles using deep image and similarity graph representations
Source: bioRxiv. 2024 Feb 8:2024.02.04.578778. Preprint. [Version 1] doi: 10.1101/2024.02.04.578778 (PMC10871265; doi:10.1101/2024.02.04.578778)
Supplement: Supplement 1 [file media-1.pdf]

Supplementary for DeepSLICEM: Clustering CryoEM particles using deep image and similarity graph representations

Meghana V. Palukuri and Edward M. Marcotte

Oden Institute for Computational Engineering and Sciences,
University of Texas, Austin, TX 78712, USA

Department of Molecular Biosciences,
University of Texas, Austin, TX 78712, USA

ORCIDs: M.V.P., 0000-0002-6529-6127; E.M.M., 0000-0001-8808-180X

Correspondence: meghana.palukuri@utexas.edu, marcotte@utexas.edu

Supplementary tables

Table S1. Different (dimensionally) reduced image embedding methods concatenated with reduced graph node embedding methods for clustering synthetic noisy images are evaluated. The results presented are the DeepSLICEM methods giving better performance than SLICEM.

Image embedding Method	Node Embedding Method	Clustering Method	Clustering parameters	No. of clusters	FMM Precision	FMM Recall	FMM F1 score	CMMF	Qi *et al.* F1 score
Siamese - noisy synthetic	Watch Your Step	OPTICS	max_eps=0.25, metric='cosine', min_samples=3	31	0.79	0.7	**0.742**	0.77	0.758
Siamese - synthetic more projections	Watch Your Step	Birch	n_clusters=None, branching_factor =50, threshold=0.8	27	0.759	0.585	0.661	0.748	0.710
Siamese - noisy synthetic	Node2Vec	OPTICS	max_eps=2.75, metric='sqeuclide an', min_samples=3	34	0.639	0.621	0.630	0.711	0.609
Siamese - synthetic more projections	Node2Vec	OPTICS	max_eps=0.25, metric='cosine'	22	0.773	0.486	0.596	0.697	0.596
EfficientNet-B7	Watch Your Step	Affinity Propagatio n	damping=0.9	22	0.755	0.474	0.583	0.693	0.456
VGG	Watch Your Step	OPTICS	max_eps=2.5, metric='braycurtis ', min_samples=3	22	0.747	0.469	0.576	0.646	0.526
EfficientNet-B7	Node2Vec	Affinity Propagatio n	damping=0.9	23	0.718	0.472	0.569	0.648	0.552
Siamese - noisy synthetic	Metapath2Vec	BIRCH	branching_factor =30, n_clusters=None	19	0.804	0.437	0.566	0.691	0.593
Siamese - synthetic	Node2Vec	Affinity Propagatio n	damping=0.9	19	0.796	0.432	0.560	0.687	0.556
VGG	Node2Vec	Affinity Propagatio n	damping=0.9	23	0.674	0.443	0.534	0.635	0.483
DenseNet	Watch Your Step	OPTICS	max_eps=2.5, metric='braycurtis ', min_samples=4	20	0.732	0.419	0.533	0.629	0.582
Siamese - synthetic	Watch Your Step	OPTICS	max_eps=0.25, metric='correlatio n',	24	0.649	0.445	0.528	0.630	0.508

			min_samples=3						
EfficientNet-B1	Watch Your Step	OPTICS	max_eps=0.5, metric='braycurtis', min_samples=4	19	0.742	0.403	0.522	0.635	0.481

Table S2. Different methods of clustering graph node embeddings using image embeddings as node attributes are evaluated for synthetic noisy images. The results presented are the DeepSLICEM methods giving better performance than SLICEM.

Image embedding Method	Node Embedding Method	Clustering Method	Clustering parameters	No. of clusters	FMM Precision	FMM Recall	FMM F1 score	CMMF	Qi *et al.* F1 score
EfficientNet-B7	APPNP	BIRCH	branching_factor=80, threshold=0.3	34	0.602	0.584	**0.593**	0.674	0.493
EfficientNet-B7	Cluster-GCN	BIRCH	branching_factor=80	34	0.579	0.563	0.571	0.637	0.406
EfficientNet-B1	Cluster-GCN	BIRCH	branching_factor=20	27	0.622	0.480	0.542	0.639	0.484
EfficientNet-B7	GraphSage	BIRCH	branching_factor=70, threshold=0.2	39	0.505	0.563	0.532	0.614	0.351
EfficientNet-B1	APPNP	BIRCH	branching_factor=60, n_clusters=None, threshold=0.2	55	0.433	0.681	0.529	0.617	0.489
